# Colorectal cancer in Crohn’s disease: a series of 6 cases

**DOI:** 10.1186/s40792-021-01237-0

**Published:** 2021-06-28

**Authors:** Kazuhide Ishimaru, Tetsuro Tominaga, Takashi Nonaka, Akiko Fukuda, Masaaki Moriyama, Shosaburo Oyama, Mitsutoshi Ishii, Terumitu Sawai, Takeshi Nagayasu

**Affiliations:** 1 Department of Surgical Oncology, Nagasaki University Graduate School of Biomedical Sciences, 1-7-1 Sakamoto, Nagasaki, 852-8501 Japan; 2 Cardiopulmonary Rehabilitation Science, Nagasaki University Graduate School of Biomedical Sciences, 1-7-1 Sakamoto, Nagasaki, 852-8501 Japan

**Keywords:** Crohn’s disease, Colorectal cancer, Surveillance

## Abstract

**Background:**

Colorectal cancer (CRC) is the most malignant complication in patients with Crohn’s disease (CD). We report 6 cases of CD-related CRC treated surgically at our hospital.

**Case presentation:**

From 2010 to 2016, six CD patients were diagnosed with CRC. All patients were diagnosed with CD at < 25 years old, and the interval from onset of CD to diagnosis of CRC was > 10 years (range, 15–42 years) in all patients. The histological type of cancer was mucinous carcinoma in two cases, well-differentiated tubular adenocarcinoma in two cases, and moderately differentiated tubular adenocarcinoma in two cases. CRC was detected by screening colonoscopy in three cases (50%), and from clinical symptoms in the remaining three cases (50%). Two cases underwent colonoscopy within 2 months after symptom onset, detecting CRC in the relatively early stage. However, one case was diagnosed with advanced-stage CRC by endoscopy 1 year after symptom onset, and experienced poor prognosis.

**Conclusions:**

Regular surveillance colonoscopy is needed to detect early-stage CRC in CD patients. Clear surveillance methods need to be established based on evidence.

## Background

Cancer is one of the most serious complications in patients with CD [1]. The risk of colorectal cancer (CRC) is reportedly higher among patients with CD than in the general population [1]. Periodic colon cancer surveillance by colonoscopy is warranted to detect CD-related CRC in the early stages. However, some CD patients show poor compliance with surveillance. Appropriate surveillance and screening methods for CRC in patients with CD still have not been clarified.

We report herein a case series of six patients who underwent surgery for CD-related CRC at our institution.

## Case presentation

From 2010 to 2016, a total of 6 CD patients underwent surgery for CRC at our hospital (Table 1). Mean patient age was 42 years (range, 35–56 years), and five patients (83%) were male. Four patients had been diagnosed with CD at < 20 years old, and disease duration ranged from 15 to 42 years. Most patients showed small intestine and colon-type CD.

**Table 1:** Six colorectal cancer patients with Crohn’s disease

Case	Age (years)	Sex	Age at diagnosis	Type of CD	Symptoms	Diagnosis methods	Tumor location	Operation	Pathology	pT	pN	pStage	Recurrence (months)	Prognosis (months)
1	35	Male	17	Colon	Defecation disorder	CS	RbP	APR	Wel	3	0	II	None	Alive (65 months)
2	48	Male	16	Small intestine and colon	None	Screening CS	PRb	APR	Mod	3	0	II	None	Alive (43 months)
3	35	Female	20	Small intestine and colon	None	Screening CS	RbP	APR	Muc	3	0	II	None	Alive (48 months)
4	42	Male	21	Small intestine and colon	Hemorrhage	CS	P	APR	Mod	Tis	0	0	None	Alive (120 months)
5	56	Male	14	Small intestine and colon	None	Screening CS	Rb	APR	Wel	2	0	I	None	Alive (22 months)
6	53	Male	16	Small intestine and colon	Anal stenosis	Tumor biopsy	RbP	APR	Muc	4	0	II	Bone (23 months)	Dead (33 months)

*CD* Crohn’s disease, *CS* colonoscopy, *APR* abdominoperineal resection, *Wel* well-differentiated type, *Mod* moderately differentiated type, *Muc* mucinous adenocarcinoma

Three patients had no clinical symptoms, and CRC was only detected on screening colonoscopy. The remaining three patients experienced clinical symptoms including defecation disorder, hemorrhage, and anal stenosis. Two of these three patients received colonoscopy within 2 months after symptom onset, but one patient refused colonoscopy due to anal pain, and cancer was detected on anal biopsy 1 year after symptom onset. All patients underwent abdominoperineal resection. Histopathological examination diagnosed mucinous cell carcinoma in two patients, well-differentiated carcinoma in two patients, and moderately differentiated adenocarcinoma in two patients. Tumors were Stage II in four patients, Stage I in one patient, and Stage 0 in one patient. Five patients experienced no recurrence. However, one patient developed multiple bone metastases and died 33 months postoperatively.

Two of the six cases (Cases 5 and 6) are described in detail.

### Case 5: a 56-year-old man

**Fig. 1:**
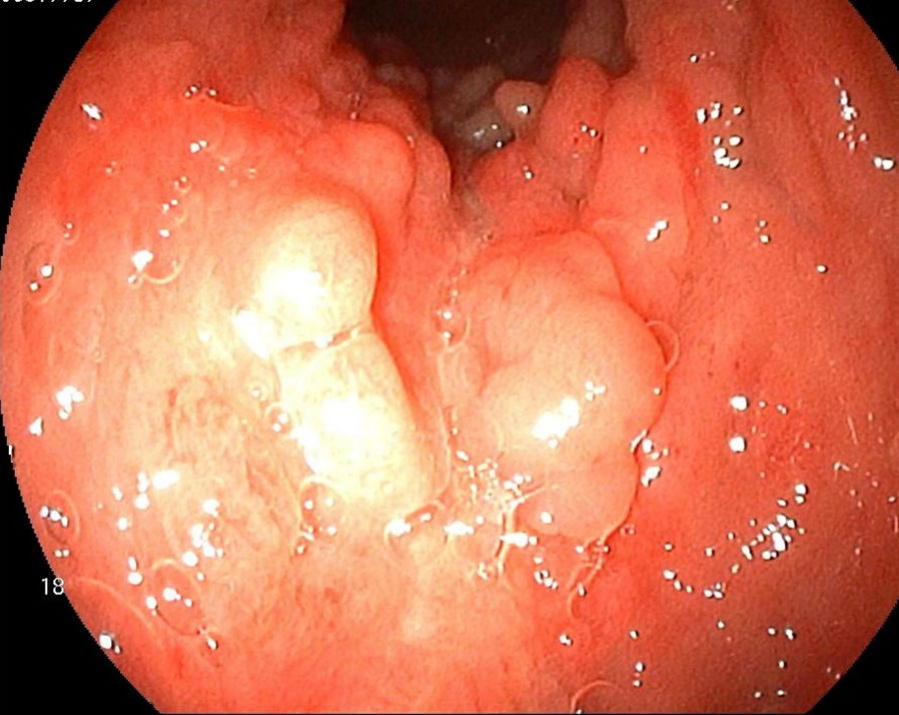
Colonoscopy shows a 0–IIb-type tumor in the lower rectum. Biopsy reveals well-differentiated adenocarcinoma

**Fig. 2:**
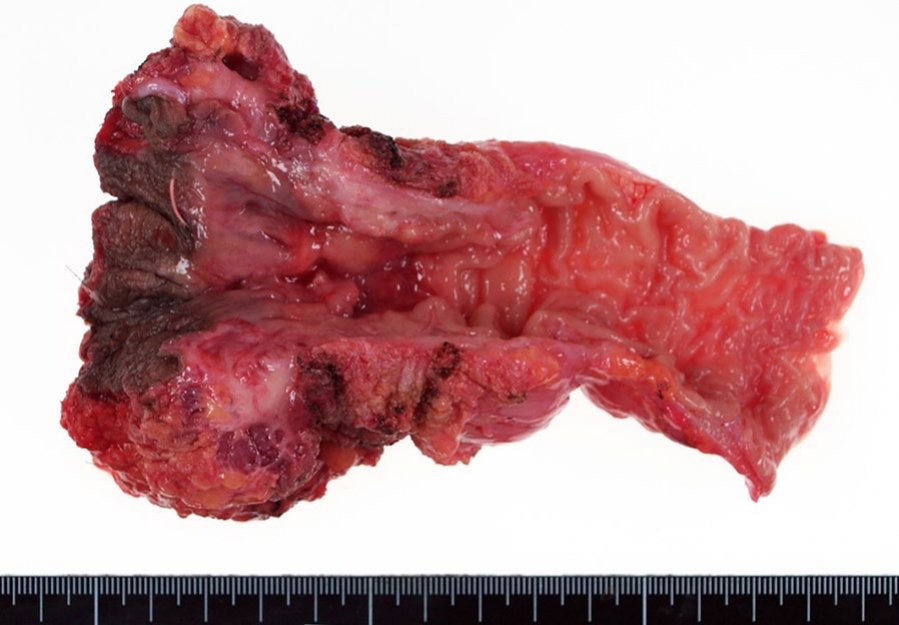
Macroscopic examination shows 0–IIb-type tumor. Tumor diameter is 20 mm

The patient was diagnosed with CD at 14 years old. He had received pharmacotherapy for 42 years. Annual screening endoscopy identified a 0–IIb-type tumor in the low rectum (Fig. 1). Pathological findings from tumor biopsy showed well-differentiated adenocarcinoma. Preoperative diagnosis was low rectal cancer, cT2N0M0 cStage I, and abdominoperineal resection was performed. Tumor diameter was 20 mm (Fig. 2). The final stage was pT2N0M0 pStage I. No recurrence has been seen as of 24 months postoperatively.

### Case 6: a 53-year-old man

**Fig. 3:**
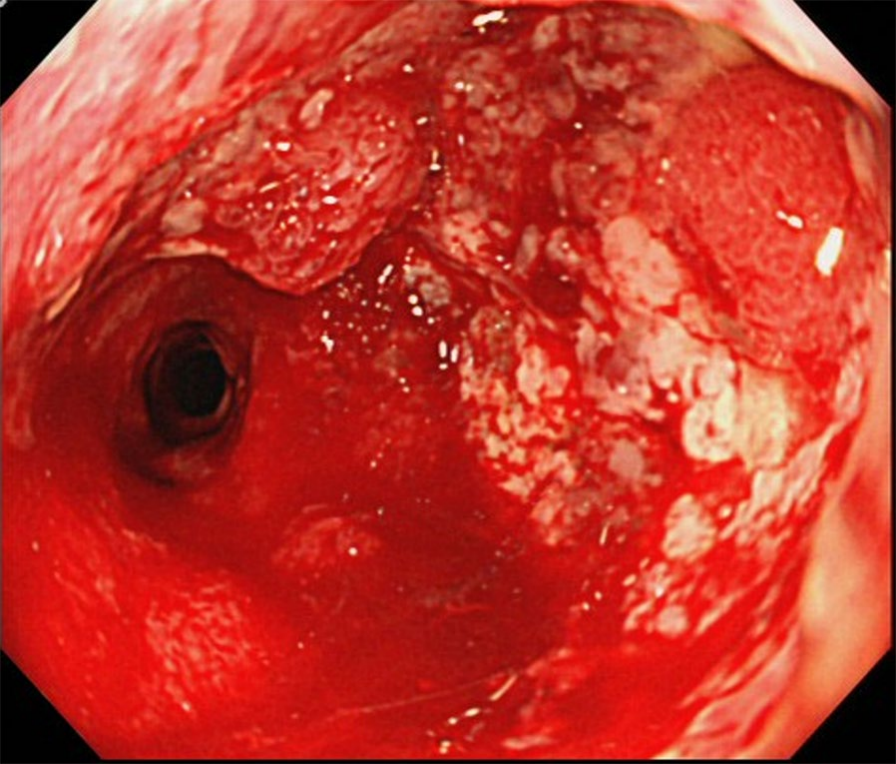
Colonoscopy shows circumferential type 2 tumor located in the lower rectum. Biopsy reveals mucinous cell carcinoma

The patient was diagnosed with CD at 16 years old, and received pharmacotherapy for 37 years. He had suffered anal stenosis, and had undergone dilation therapy by endoscopy several times. One year before surgery, continuous anal stenosis developed. However, he showed poor compliance with endoscopic examination due to busyness and anal pain. One year after occurrence of clinical symptoms, the anal tumor was detected (Fig. 3). Finally, mucinous adenocarcinoma was diagnosed from a tumor biopsy.

**Fig. 4:**
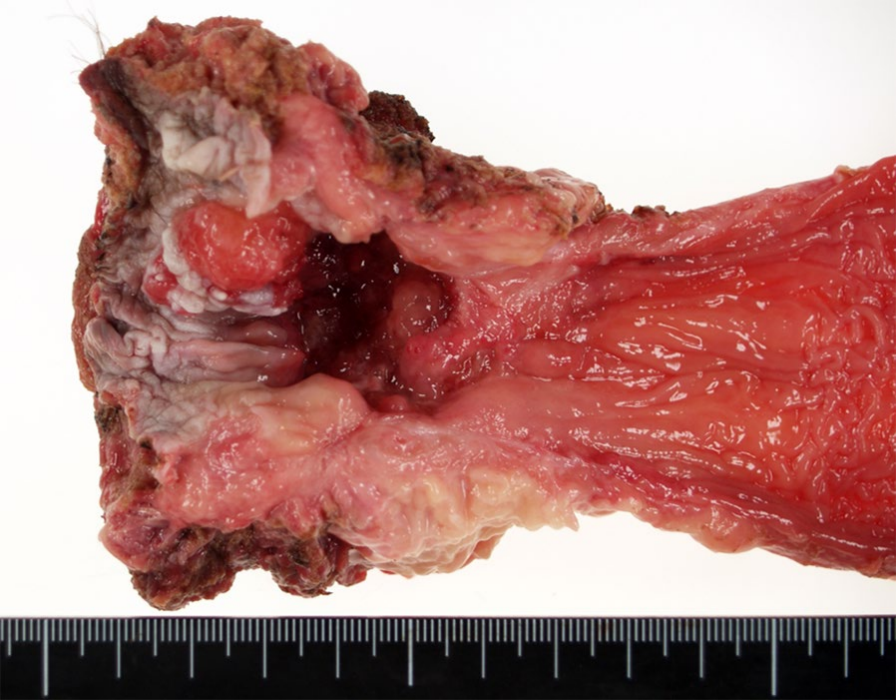
Macroscopic examination shows type 2 tumor located in the lower rectum. Tumor diameter is 40 mm

Preoperative diagnosis was low rectal cancer, cT4N0M0 cStage II, and abdominoperineal resection was performed. Tumor diameter was 40 mm (Fig. 4). Postoperative adjuvant chemotherapy (UFT: tegafur–uracil) was administered. Multiple bone metastases were detected at 23 months postoperatively, and systemic chemotherapy therefore initiated, but he died 33 months after surgery.

## Discussion

CD is an inflammatory bowel disease that can develop in any segment of the gastrointestinal tract from the mouth to the anus. An epidemiological study estimated that over 70,000 people (55.6 per 100,000 people) in Japan have CD [2].

CD has the potential to increase the risk of CRC due to long-term chronic inflammation via a dysplasia–carcinoma sequence [3]. Risk factors for CRC associated with CD are younger age of CD diagnosis and longer period of observation [4–8]. Scaringi et al. reported that CD patients who require surgery are at higher risk of developing CRC, particularly those with disease duration > 10 years, distal localization, age at diagnosis < 40 years, and penetrating disease [9]. Yano et al. also found that the cumulative incidence of CRC in 512 CD patients increased over time (0.25% at 10 years, 0.6% at 15 years, and 0.6% at 20 years) and was higher than in the general population [4]. In our cases, the age of CD onset was < 25 years old in all cases, and the interval from onset of CD to diagnosis of CRC was > 10 years in all patients, and > 20 years in four cases.

Ikeuchi et al. studied 504 CD patients and found nine patients with carcinoma, with 77% in the lower rectum and anus with severe anorectal CD lesions [10]. The cumulative 5-year survival rate for CRC was only 46.2%, worse than that for the general population [11]. In addition, CD-related CRC shows a high rate of mucinous carcinoma and Signet-ring cell carcinoma [1, 12, 13]. In this case series, two patients (33%) were diagnosed with mucinous adenocarcinoma.

Considering these clinical and pathological data, early detection by surveillance is crucial. However, CD patients have a high rate of small bowel cancer and anal canal cancer with severe anorectal CD lesions, increasing the difficulty of performing colonoscopy because of the associated anorectal stenosis or pain. Effective surveillance methods that are linked to the prognosis of CD-related gastrointestinal cancers are currently lacking [4, 14, 15]. Hirano et al. conducted surveillance for 103 of 116 patients after excluding 13 patients diagnosed with cancer-related symptoms, and found cancer and atypical cells in 6 patients (5.8%) [15]. They also reported that patients in whom cancer was detected without symptoms showed better prognosis than those detected based on symptoms, according to survival curves. Friedman et al. emphasized the importance of annual surveillance endoscopy [16]. Indeed, CD patients with rectal or anal lesions experience difficulty with endoscopy, and 23% of surveillance examinations in this group reportedly required a small diameter colonoscope due to stenosis. However, annual colonoscopy helped to detect early-stage neoplasm. In our cases, three patients were detected early by annual surveillance endoscopy. The remaining three patients were detected by clinical symptoms. Fortunately, two cases received colonoscopy within 2 months after symptom onset, detecting CRC in the relatively early stage with preferable prognosis. However, one patient was diagnosed with advanced-stage CRC by endoscopy 1 year after symptom onset, and experienced poor prognosis.

### Limitations

Some limitations to this study must be considered. First, the number of cases was small, so verification of appropriate follow-up methods must be carried out by accumulating cases in the future. Second, these six cases had a long-term course, and the current medical treatment of CD has changed from that used in these cases. Development of surveillance methods based on the present medical treatment of CD is therefore necessary.

## Conclusions

For patients with CD, CRC needs to be detected at a relatively early stage by performing surveillance endoscopy regularly or by performing examination as soon as symptoms appear.

## Abbreviations

CD: Crohn’s disease
CRC: Colorectal cancer
